# Platform process will give platform product - Can we afford it?

**DOI:** 10.1186/1753-6561-7-S6-P88

**Published:** 2013-12-04

**Authors:** Rohit Diwakar, Sunaina Prabhu, Lavanya C Rao, Janani Kanakaraj, Kriti Shukla, Saravanan Desan, Dinesh Baskar, Ankur Bhatnagar, Anuj Goel

**Affiliations:** 1 Cell Culture Lab, Biocon Research Limited, Bangalore, India

## Introduction

Manufacturing processes for therapeutic monoclonal antibodies (mAbs) have evolved immensely in the past two decades around two major thrust areas.

1) Advancements in a) Cell line development-breakthrough and incremental knowledge gain in technology b) Media and feed formulation strategies c) Advent of Disposables and Instrumentation technologies thus offering significant improvements to Process Development (PD).

2) Establishment of platform processes to leverage faster PD [1,2].

A platform process generally consists of a standard i) Cell line development technique, ii) Basal medium and feeds, iii) Process parameters and scale-up approach. The biggest advantage of using the platform process for the PD group is in expediting the project timelines. The platform approach also benefits from well-established and validated work flows in Manufacturing, QA, QC and Supply-chain groups.

Certain disadvantages have also been cited for the platform approach. For example, modifications in the platform process are generally discouraged due to time, cost and efforts required in accommodating such changes. Also, as process conditions can substantially impact the product quality (PQ) attributes, a platform approach does not allow any significant changes in the PQ attributes, if desired.

## Materials and methods

In this study, CHO cell lines were cultured in chemically defined medium. Experiments were carried out in 2L stirred tank bioreactors and 125mL shake flasks running at 140 rpm in 5% CO_2 _controlled incubator shaker. Cell count and viability were determined using haemocytometer. Lactate, glucose, osmolality and IgG concentration was also estimated along with glycosylation profiling.

## Results and discussion

### Case 1: Multiple cell lines developed using same technology expressing different mAbs

Using the same cloning technology, cell lines expressing mAbs 1-4 were developed. These cell lines when run with the platform process showed very similar growth, titer and glycosylation profiles. Glycan profile thus produced is represented as three species; type I, II and III.

The advantage of platform process was evident from the similarity of glycan profiles achieved in all the mAbs run with this process. However, for mAbs 3 and 4, the target glycan profile was significantly different. The platform process gave 20-30% higher glycan type 1 than the respective targets. In order to match the targeted glycan profile, a few changes were made:

i) mAb 3: New feed introduced to reduce glycan type 1; feeding strategy was optimized during PD.

ii) mAb 4: In addition to feeding strategy used for mAb3, changes in process parameter (pH and DO) set-points were done to achieve desired glycosylation profiles.

### Case 2: Difference in lead clone selection criteria - growth vs. specific productivity

Clone selection is done by ranking the clones based on parameters such as cell growth, titer, specific productivity (PCD) and PQ. In this study, the lead clones were shortlisted based on different strategies. For mAbs 1-4, the lead clone was shortlisted based on cell growth and titer as dominant selection criteria. For mAbs 5 and 6, PCD was the dominant selection criterion. The other aspects of the cloning technique were same in all cell lines.

**Figure 1:**
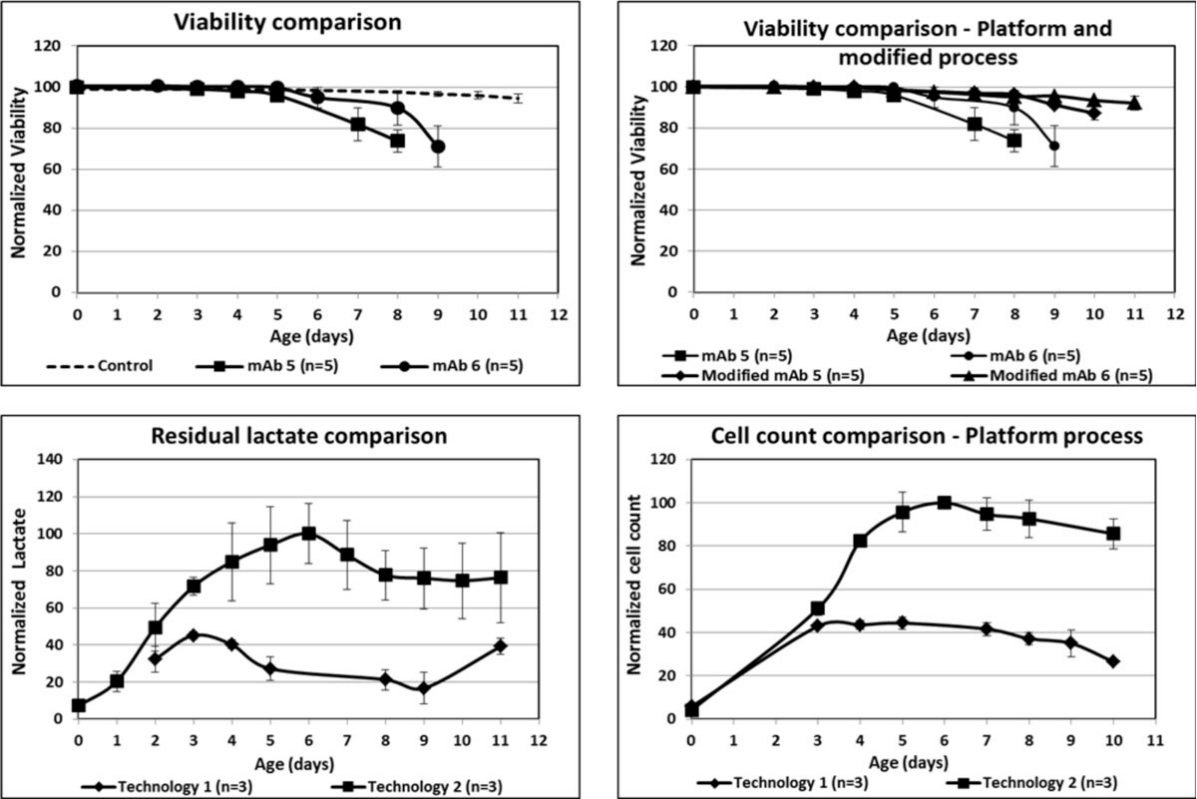
**(Clockwise direction) a) Viability comparison between control (mAb1-4) and mAb5 and 6**. **b) **Viability comparison between platform and modified process for mAb5 and 6. **c) **Cell count comparison and **d) **Lactate comparison between cell line technology 1 and 2. As expected, PQ profiles between these two clones were very different.

When lead clones for mAb 5 and 6 were run in platform process they showed poor growth characteristics (Figure 1a). The early drop in viability made these clones unfit for a manufacturing process. Changes in the platform process were attempted to overcome this manufacturing concern:

i) mAb 5: Culture longevity was increased by restricting cell growth. This was achieved by reducing nutrient levels in the production medium.

ii) mAb 6: Lactate and ammonia accumulation was reduced by optimizing medium/feed composition and pH, DO control ranges.

The modified processes significantly improved the culture longevity and viability profiles, making them suitable for manufacturing (Figure 1b).

### Case 3: Cell lines expressing the same mAb developed using different technology

Two cloning technologies, 1 and 2 were used to develop clones expressing the same mAb. The major differences in the technologies were i) host cell lines ii) design of vector and its mechanism in the genome. Both cell lines were run with the same platform process and a two-fold difference in cell count was observed between them (Figure 1c). The lactate levels were also markedly different (Figure 1d), possibly indicating differences in nutrient metabolism. The lactate differences also reflected in the pH profiles.

## Summary

Case 1: The use of platform process enabled accelerated PD from cell culture perspective. However, accommodating the specific PQ requirements resulted in extended process development, affecting timelines.

Case 2: Change in clone selection criteria was observed to significantly impact culture performance while applying platform process. This almost resulted in rejection of these clones, thus extending PD timelines. This was prevented by modifying the platform process.

Case 3: Clones developed using different cloning technologies when run with the platform process resulted in different cell culture and PQ profiles. Therefore, the type of cloning technique forms an integral part of the platform process.

Though platform process was not suitable in most of the cases discussed here, it still offers advantages like expedited project timelines and established work flows. These benefits were achieved by establishing four versions of the platform process to meet the varied cell culture and PQ requirements. Based on the cell line characteristics and target PQ profiles, the appropriate version is chosen to initiate PD. These versions retained the major advantages of the platform process such as having common media and feeds with only changes in their concentrations and set point of main process parameters to achieve desired PQ.
